# Harnessing Organometallic Au(III) Complexes as Precision Scaffolds for Next‐Generation Therapeutic and Imaging Agents

**DOI:** 10.1002/cbic.202500347

**Published:** 2025-06-04

**Authors:** Sophie R. Thomas, Riccardo Bonsignore

**Affiliations:** ^1^ Department of Inorganic Chemistry University of Vienna Währinger Straße. 42 1090 Vienna Austria; ^2^ Dipartimento di Scienze e Tecnologie, Biologiche, Chimiche, e Farmaceutiche Università degli Studi di Palermo 90128 Palermo Italy

**Keywords:** bioinorganic catalysis, C–S cross‐coupling, cyclometalated Au(III) complexes, cysteine arylation, metal‐based therapeutics

## Abstract

Au(III) organometallic complexes, particularly cyclometalated Au(III) compounds, have emerged as powerful tools in catalysis and bioinorganic chemistry, offering unique reactivity distinct from their Au(I) counterparts. Among their most interesting transformations, C–S cross‐coupling reactions have become a selective strategy for cysteine arylation, enabling site‐specific modifications of peptides and proteins. This review provides a comprehensive overview of cyclometalated Au(III) complexes in C–S bond formation, detailing the mechanistic insights, ligand effects, and electronic factors that dictate their reactivity. The role of ancillary ligands in tuning stability and selectivity is critically assessed, alongside advancements in structural modifications that enhance catalytic efficiency. Beyond fundamental C–S cross‐coupling, the broader applications of these Au(III) complexes are explored, including enzyme inhibition, metabolic disruption, and transmembrane protein modulation, with implications in anticancer therapy, antimicrobial strategies, and in vivo catalytic transformations. By bridging fundamental organometallic reactivity with innovative biomedical applications, this review highlights the potential of cyclometalated Au(III) complexes as next‐generation catalysts for both synthetic and therapeutic innovations.

## Graphical abstract

Cyclometalated gold(III) complexes offer a versatile platform for selective biological interactions, including cysteine arylation, redox modulation, and enzyme inhibition. This review explores their roles in anticancer, antimicrobial, and protein modification strategies, highlighting their ability to disrupt metabolic pathways, modulate aquaporins, and serve as precision tools for next‐generation therapeutics and imaging applications.

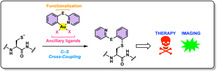

## 1 Introduction

The use of gold in medicine dates back to the early centuries when it was used in traditional medicine as a treatment for disease to defend against evil spirits.^[^ ^1^ ^]^ Pioneering work in the late 19th century by the German physician Robert Koch, who showed the therapeutic ability of K[Au(CN)_2_] against tuberculosis,^[^ ^2^, ^3^ ^]^ led to the expansion of gold therapeutics in the modern era. Since then, polymeric Au(I) thiolate complexes, including aurothiomalate and aurothioglucose, found use in the clinic as anti‐arthritis agents, paving the way for the discovery of auranofin, which showed higher activity with less severe side effects.^[^ ^4^, ^5^ ^]^ Auranofin has also been involved in drug‐repurposing trials for the treatment of different types of cancer due to its acute toxicity in vitro, and even in platinum‐resistant cancer cell lines.^[^ ^6^ ^]^ Investigations into the mechanism of action of auranofin and other Au(I) families of complexes revealed their high affinity for selenol groups, such as those found in thioredoxin reductase (TrxR), which can be explained by the hard‐soft acid‐base (HSAB) theory.^[^ ^7^, ^8^ ^]^ However, despite these properties, Au(I) complexes remain limited in the clinic and often suffer from scarce solubility and stability, especially in physiological media, due to the fast speciation of labile ligands resulting in the formation of Au^0^ with unpredicted side effects. Nevertheless, N‐heterocyclic (NHC) ligands have been explored to overcome this due to the formation of a strong organometallic bond. As such, Au(I) organometallic complexes have gained wide attention from the scientific community for their capability to interact with DNA structures^[^ ^9^, ^10^, ^11^, ^12^ ^]^ or for their antibacterial and antiparasitic effects.^[^ ^13^, ^14^, ^15^ ^]^ However, as these families of compounds are out of the scope of the present manuscript, we address the reader to other detailed reviews on the topic.^[^ ^16^, ^17^, ^18^, ^19^ ^]^

Investigations with Au(III) complexes to bypass the resistance and toxicity observed for Pt(II) anticancer complexes (e.g., cisplatin) should also be highlighted since Au(III) is isoelectronic and isostructural to Pt(II). However, unlike cisplatin, which is well‐known for its ability to bind to DNA bases such as guanine, Au(III) complexes typically follow a mechanism of action involving direct coordination to soft amino acids within enzymes or proteins, that is, cysteines (Cys), methionines, and selenocysteines (Sec). Nevertheless, Au(III) complexes often experience stability issues due to the high oxidant character of Au(III), which allows the facile metal reduction to the +1 oxidation state. To circumvent this problem, several researchers have focused on bi‐, tri‐, and tetra‐dentate chelating ligands involving coordination bonds from the Au(III) to nitrogen, oxygen, sulfur, or phosphorus atoms. Despite such ligand‐chelating effects increasing the stability of the final complex, it is often not enough to guarantee the compounds’ integrity in physiological media.

Therefore, to address this challenge, cyclometalating ligands have become an attractive alternative, offering improved stability due to the formation of an organometallic Au−C bond. A variety of different cyclometalating ligands can be used with different donor atoms (e.g., N‐, O‐, S‐, Se‐, or P‐), with nitrogen being the most commonly explored for Au(III) complexes due to the HSAB theory. Within the different donor atoms, the combination of different binding sites is also possible leading to bidentate (e.g., C^N or C^C) or tridentate (e.g., C^N^C, N^C^N, and C^N^N) ligands. This increased stability has led to their use in many applications, including light‐emitting compounds in organic light‐emitting diodes (OLEDs),^[^ ^20^, ^21^ ^]^ catalysts,^[^ ^22^, ^23^, ^24^, ^25^ ^]^ and anti‐tumor^[^ ^26^, ^27^, ^28^ ^]^ and anti‐leishmanial^[^ ^13^, ^29^ ^]^ drug molecules. For a more comprehensive overview of the synthesis and reactivity of such complexes, the reader is directed to further reviews.^[^ ^30^, ^31^ ^]^

## 2 C–S Cross‐Coupling Reactions

Another area of interest is the ability to template bioconjugation reactions with amino acids, specifically the arylation of Cys residues. Cys is the only thiol‐containing canonical amino acid and, thus, is relatively rare in the human genome. Furthermore, the Cys thiol/thiolate group (pKa ≈ 8 in aqueous media) is significantly more nucleophilic than other amino acid side chains, making it highly reactive toward electrophiles, which enables chemoselective targeting.^[^ ^32^ ^]^ Intriguingly, although it has been known for decades that organic compounds such as haloacyl‐compounds and maleimides can arylate Cys residues *via* nucleophilic aromatic substitution (S_N_Ar), the cross‐reactivity of these compounds with other amino acid side chains has resulted in the drive for more chemoselective alternatives.^[^ ^32^ ^]^

**Figure 1:**
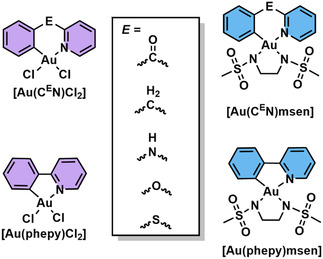
Structures of cyclometalated Au(III) organometallic complexes highlighted in this review.

Transition metal complexes have been known to mediate C–S cross‐coupling reactions within organic solvents for small molecule synthesis over four decades;^[^ ^33^ ^]^ however, it wasn't until 2014 that the first example of transition metal catalysts to template the C–S cross‐coupling reaction with a biomolecule was published.^[^ ^34^ ^]^ In this work, several Au(III) cyclometalated complexes with different bridging ligands (E = CH_2_, CO) and *N*,*N*′‐bis(methanesulfonyl)ethylenediamine) (msen) as ancillary ligand ([Au(C^CH2^N)msen] and ([Au(C^CO^N)msen], C^CH2^N =2‐benzylpyridine and C^CO^N = 2‐benzoylpyridine, **Figure** **1**) were studied for their ability to arylate short Cys‐containing peptides. This seminal work demonstrated the formation of Cys arylation products in phosphate‐buffered saline (PBS, pH 7.4) with dimethyl sulfoxide (DMSO) as co‐solvent (9:1, respectively) at room temperature over 2 h. Interestingly, the ability of the 5‐membered [Au(phepy)msen] (phepy = 2‐phenylpyridine) complex was tested in the same conditions, showing no Cys arylation product but the formation of a Cys coordination adduct, whereby the Au(III) center is bound to the peptide with loss of the msen ligand. The Cys arylation scope was further expanded with successful C–S bond formation with human serum albumin (HSA), which contains a single exposed Cys on its surface.^[^ ^34^ ^]^

Such a result is of particular importance for the potential application in biological systems. For instance, zinc fingers (ZFs) are proteins that possess cysteines or a combination of cysteine and histidine ligands around the Zn(II) center in a tetrahedral geometry.^[^ ^35^ ^]^ The nature of the ligand system is responsible for the role and structure of the ZF, which in turn allows it to interact with sequence‐specific sections of DNA, RNA, poly‐ADP‐ribose (PAR), and other proteins, depending on their function.^[^ ^35^ ^]^ Additionally, due to their role in DNA transcription, translation, and repair, they have been associated with cancer progression.^[^ ^36^ ^]^ Therefore, the ability of Au(III) complexes to irreversibly modify the Cys residues could inhibit the overall ZF function.

In 2018, Farrell and coworkers demonstrated the ability of the [Au(C^CH2^N)Cl_2_] complex (Figure 1) to perform Cys arylation with the Cys of the HIV NCp7 ZF nucleocapsid protein.^[^ ^37^ ^]^ This was observed by high‐resolution electrospray ionization mass spectrometry (HR‐ESI‐MS), where an equimolar amount of the gold complex was incubated with the ZF core. At first, the coordination complex was observed (apo‐ZF‐[Au(III)C^CH2^N]), but this was then followed by the Cys arylation product (apo‐ZF‐[C^CH2^N]) after 48 h incubation. Furthermore, C–S bond formation was also demonstrated with the ZnCys_2_His_2_ coordination sphere of the Sp1 transcription factor.^[^ ^37^ ^]^

## 3 Mechanistic Insights

**Scheme 1:**
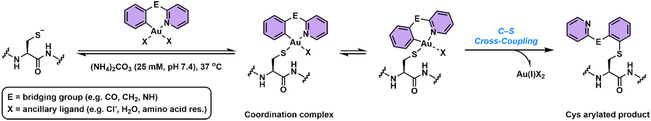
Proposed mechanism of Cys arylation templated by Au(III) cyclometalated complexes.^[^ ^38^ ^]^

To investigate the effect of different bridging groups (E) on the Cys arylation ability of Au cyclometalated complexes ([Au(C^E^N)Cl_2_]), Casini and coworkers explored the difference in reactivity of the complexes with different bridging groups, E = CO, CH_2_, and NH ([Au(C^CO^N)Cl_2_], [Au(C^CH2^N)Cl_2_] and [Au(C^NH^N)Cl_2_], C^NH^N = *N*‐phenylpyridin‐2‐amine), as well as the 5‐membered [Au(phepy)Cl_2_] complex, with a model peptide sequence of Cys_2_His_2_ ZF domain (Figure 1).^[^ ^38^ ^]^ In this work, 3 eq. of the Au complexes were incubated with the ZF peptide in ammonium carbonate buffer (25 mM, pH 7.4, 37 °C), with measurements taken at 10 min and 24 h. HR‐LC‐ESI‐MS (LC = liquid chromatography) was used to detect the formation of the classical coordination complex (apo‐ZF‐[Au(III)C^E^N]) and the Cys arylated product (apo‐ZF‐[C^E^N]). After 10 min, all three of the six‐membered [Au(C^E^N)Cl_2_] complexes formed coordination adducts, with the CO bridging complex also demonstrating Cys arylation after this short time, revealing remarkable reactivity. After 24 h, all three complexes yielded both coordination and arylation products. However, even after 24 h, [Au(phepy)Cl_2_] could only form coordination adducts. These reactivity differences were further explored by density functional theory (DFT) calculations, where a reaction mechanism was proposed involving the substitution of at least one chloride ligand with a Cys residue before the apical approach of a second Cys, which could bind to the gold center and replace the N of the C^N ligand. This intermediate could then react further, resulting in the reductive elimination of the Au(III) as Au(I) and the formation of the C–S bond (**Scheme** **1**).^[^ ^38^ ^]^

Probing further into the reactivity of such gold complexes for Cys arylation, the authors investigated two other ([Au(C^E^N)Cl_2_]) (E = O and S, [Au(C^O^N)Cl_2_] and [Au(C^S^N)Cl_2_], C^O^N = 2‐phenoxypyridine, C^S^N = 2‐(phenylthio)pyridine, Figure 1) complexes alongside the highly reactive [Au(C^CO^N)Cl_2_], as well as a tridentate C^N^N Au(III) complex ([Au(bipy^dmb^H)Cl]PF_6_) (bipy^dmb^ = 6‐(1,1‐dimethylbenzyl)‐2,2’‐bipyridine).^[^ ^39^ ^]^ Using the same mass spectrometry approach and Cys_2_His_2_ model peptide as in previous work,^[^ ^38^ ^]^ all four complexes formed the classical coordination adduct due to the substitution of one or two chlorides.^[^ ^39^ ^]^ However, even after 24 h, only the three AuC^N complexes were able to form the Cys arylation product adducts, with [Au(C^CO^N)Cl_2_] being the most reactive,^[^ ^39^ ^]^ further highlighting that the presence of a cyclometalating ligand was essential for the C–S cross‐coupling. In addition, the chemoselectivity of the [Au(C^E^N)Cl_2_] complexes was investigated with two other peptides containing a single Cys residue, positioned either in the middle (ANGELACASINI (AC)) or at the end (CASINI) of the sequence. All three Au(III) organometallic complexes induced Cys arylation on the AC peptide, while no C–S bond formation was observed with the CASINI peptide. DFT studies concluded that secondary binding sites on the peptide were required to template the Cys arylation reaction, aiding reductive elimination, which were not found in the CASINI sequence. Intriguingly, these coordinating sites could also be other amino acid residues other than Cys, broadening the application of the AuC^N‐templated C–S cross‐coupling reactions. Furthermore, with all peptide sequences, a tridentate Au^III^C^N^N complex could only form the coordination adduct, emphasizing the ligand's role in the mechanism of Cys arylation.^[^ ^39^ ^]^

## 4 Other Au(III) Organometallic Complexes

**Figure 2:**
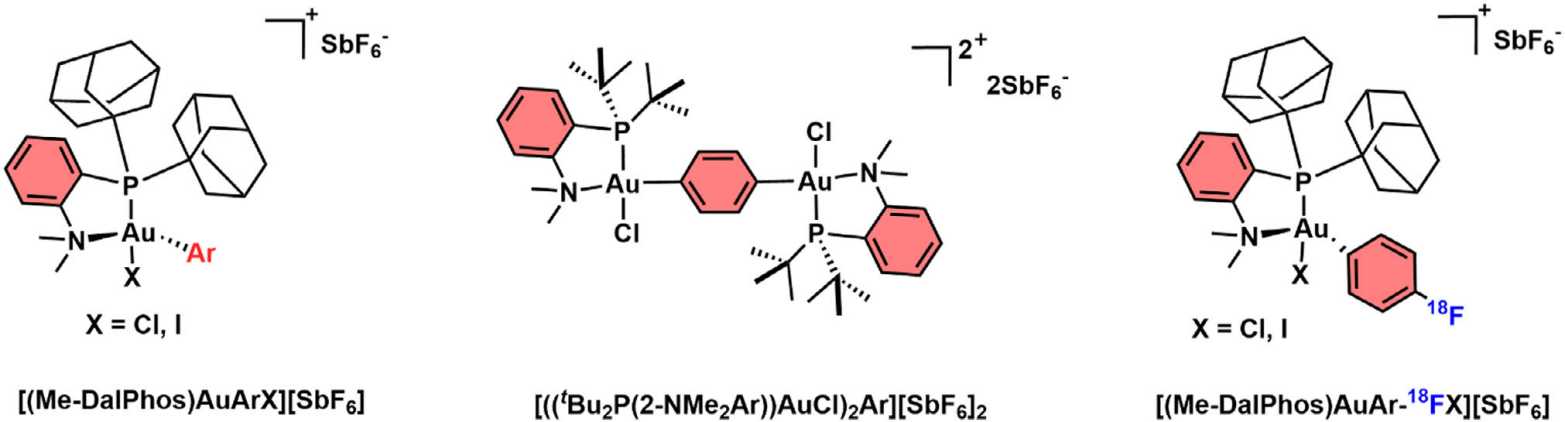
Structures of organometallic Au(III) complexes used by Spokoyny and coworkers for the arylation of cysteine residues.^[^ ^40^, ^41^, ^42^ ^]^

Notably, further Au(III) complexes, other than Au(III) C^N cyclometalated ones, can template Cys arylation reactions. In 2018, Spokoyny and coworkers published seminal work for the arylation of Cys residues with an oxidative addition complex, [(Me‐DalPhos)AuArX][SbF_6_] (Me‐DalPhos = Ad_2_P(*o*‐C_6_H_4_)NMe_2_; Ad = adamantyl; Ar = aryl, X = Cl or I, **Figure** **2**).^[^ ^40^ ^]^ In this work, multiple aryl ligands were tested with glutathione (GSH) as the model Cys for arylation, which was monitored using LC‐MS. The reaction was also carried out in more complex systems, such as DARPin (designed ankyrin repeat protein) and FGF2 (fibroblast growth factor 2), both of which have at least one accessible Cys residue,^[^ ^40^ ^]^ showing the versatility and biocompatibility of such reactions and their selectivity towards sulfur‐containing residues.

The authors also extended this work to form hybrid peptide‐based assemblies whereby the use of an aryl‐bridged bimetallic complex, [((^*t*^Bu_2_P(2‐NMe_2_Ar))AuCl)_2_Ar][SbF_6_]_2_ (^*t*^Bu_2_P(2‐NMe_2_Ar = 2‐(di‐*tert*‐butylphosphino)dimethylaminobenzene; Ar = aryl, Figure 2) was used to produce peptide staples with two Cys residues.^[^ ^41^ ^]^ Such peptide staples exhibit some advantages to their linear counterparts, such as increased cell permeability and high stability toward proteolytic degradation. The scope was further expanded to yield peptide bicycle systems through trimetallic Au(III) complexes with a central aryl motif and multisite peptide conjugation with three independent glutathione molecules, demonstrating the versatility of site‐specific Cys modifications and how these can be useful for achieving peculiar peptide properties.^[^ ^41^ ^]^

Further work by Spokoyny and coworkers involved the radiochemical labeling of Cys residues using the same Au(III) organometallic complex as in previous work but with the addition of an ^18^F‐aryl group ([(Me‐DalPhos)AuAr‐^18^FX][SbF_6_], Figure 2).^[^ ^42^ ^]^ This allowed the ^18^F‐labeling of unprotected peptides and sugars in aqueous media, enabling the rapid synthesis of ^18^F‐labeled S‐aryl bioconjugates under mild conditions. The chemoselective approach capitalizes on the electrophilicity of Au(III) complexes, allowing efficient Cys‐arylation in an aqueous environment within 15 min, with high radiochemical yields and excellent functional group tolerance.^[^ ^42^ ^]^ The stability and rapid reactivity of these gold‐based reagents highlight their promise for bioorthogonal labeling in radiopharmaceutical applications, expanding the role of Au(III) complexes beyond catalysis, demonstrating their utility in biochemical tracking, molecular diagnostics, and targeted imaging of disease‐related biomolecules.

## 5 Biological Applications

Building on these initial insights into the unique reactivity of gold organometallic complexes, recent advances have propelled Au(III) compounds to the forefront of bioinorganic chemistry research. In particular, by exploiting mechanisms such as Cys arylation, Au(III) organometallic complexes are now being tailored not only as biochemical probes but also as potent agents in antimicrobial and anticancer strategies. A prime example is the use of cyclometalated Au(III) C^N complexes as antimicrobial agents. The study by Chakraborty et al. highlights the potent activity of the [Au(C^CH2^N)Cl_2_] complex (Figure 1) against Gram‐positive bacteria, including methicillin‐resistant *Staphylococcus aureus* (MRSA) and *Bacillus subtilis*.^[^ ^43^ ^]^ This gold complex interferes with bacterial metal homeostasis by targeting thioredoxin reductase (TrxR), a key enzyme in bacterial redox balance, disrupting bacterial defense mechanisms against oxidative stress, ultimately leading to cell death. Furthermore, transcriptional analysis revealed upregulation of genes involved in metal ion transport, suggesting that the Au(III) complexes perturb essential bacterial metabolic pathways. On the contrary, ex vivo studies on treated mice tissues revealed scarce toxicity for the organometallic complex, reinforcing its potential application as a drug.

The selectivity of Au(III) complexes for Cys residues also extends to bacterial proteomes. Hacker and coworkers employed a competitive chemoproteomic profiling approach to identify ligandable cysteines in *S. aureus* targeted by the cyclometalated Au(III) complex, [Au(C^CO^N)Cl_2_] (Figure 1).^[^ ^44^ ^]^ More than 100 ligandable sites were discovered, many of which were previously inaccessible to classical organic electrophiles, underscoring the unique reactivity of Au(III) complexes. The study also confirmed the selective inhibition of bacterial TrxR, providing further evidence of the role that gold complexes play as selective modulators of bacterial (and mammalian) redox systems.^[^ ^44^ ^]^

**Figure 3:**
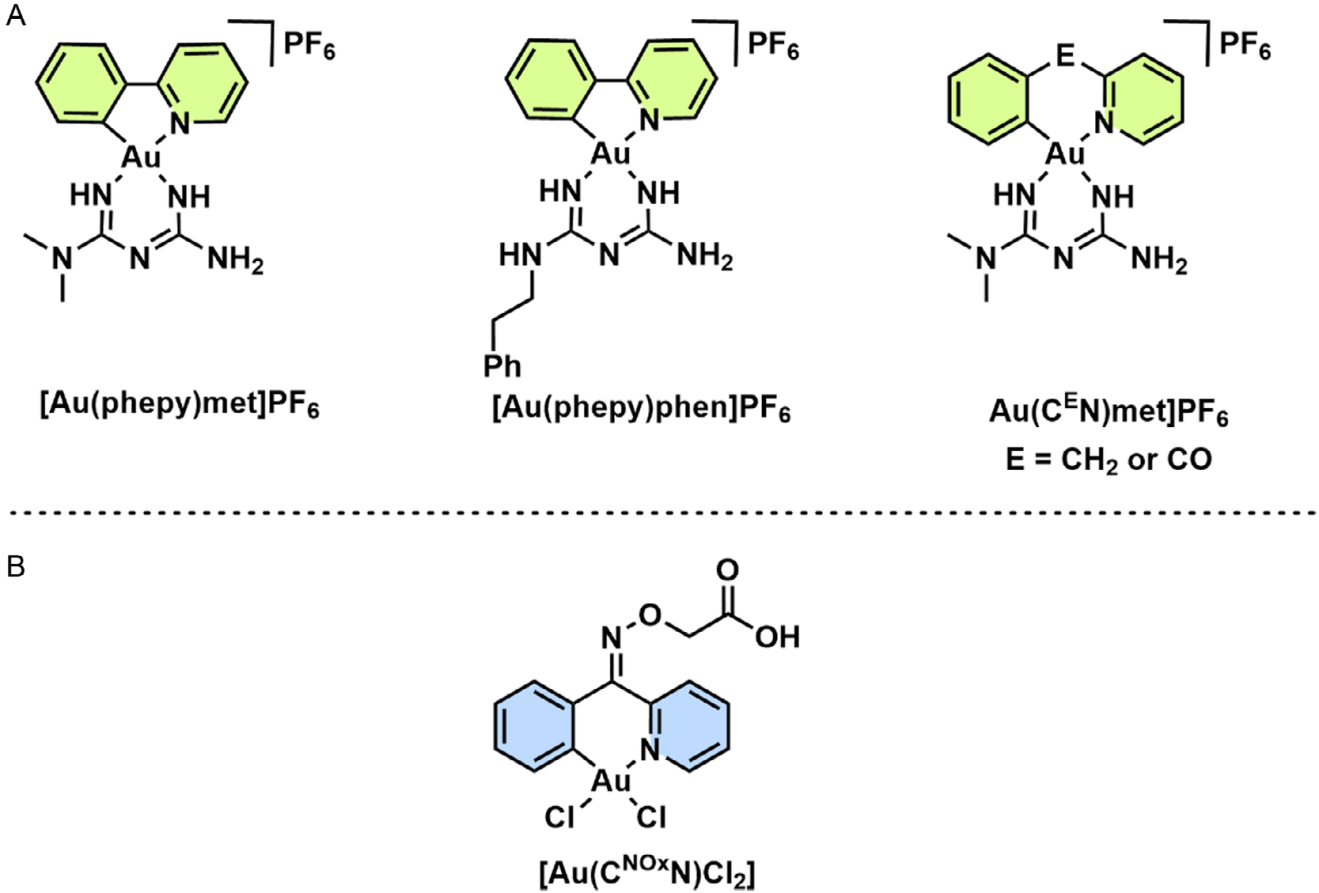
Structures of Au(III) C^N cyclometalated complexes bearing A) metformin or phenformin as ancillary ligands,^[^ ^45^ ^]^ and B) carboxylic acid as an anchoring group for proteomic studies.^[^ ^46^ ^]^

Such ability of Au(III) complexes to target redox‐sensitive pathways extends into cancer therapy, particularly in disrupting metabolic homeostasis in aggressive tumor cells. Babak et al. developed a series of triple‐negative breast cancer (TNBC) targeting Au(III)‐cyclometalated prodrugs with phepy, C^CO^N or C^CH2^N ligands bearing either metformin (met) or phenformin (phen) as ancillary ligand (**Figure** **3**A).^[^ ^45^ ^]^ These compounds effectively inhibited mitochondrial oxidative phosphorylation, leading to ATP depletion, 5′‐adenosine monophosphate‐activated protein kinase (AMPK) activation, and mTOR suppression, ultimately triggering a metabolic cascade in cancer cells. In particular, the lead complex, [Au(C^CO^N)met]PF_6_, exhibited remarkable cytotoxicity, 6000‐fold more than uncoordinated metformin. The same compound was also highly reactive towards GSH, which the authors postulated was due to loss of the metformin ligand and subsequent Cys arylation, although this was not explored further. Notably, the mode of action involved TrxR inhibition, reinforcing a mechanistic overlap between gold‐based antibacterial and anticancer strategies. Additional investigations revealed that these Au(III) complexes selectively accumulate in cancerous mitochondria, further impairing mitochondrial dysfunction and disrupting cellular respiration. This, in turn, resulted in increased reactive oxygen species (ROS) production, driving cancer cell apoptosis.^[^ ^45^ ^]^ These findings are particularly relevant given the ongoing interest in mitochondria‐targeted therapies, positioning Au(III)‐based prodrugs as potential candidates for metabolically targeted cancer treatment.

In order to understand the degree of selectivity these complexes have for Cys or Sec residues in the human cell, Meier‐Menches and coworkers used advanced chemoproteomic approaches to investigate the binding of a Au(III)C^N cyclometalated complex, [Au(C^NOx^N)Cl_2_] (C^NOx^N = 2‐(phenyl‐(2‐pyridinylmethylene)aminoxy acetic acid, Figure 3B), with the CysSec‐dyad of thioredoxin reductase 1 (TXNRD1) in human SW480 colon cancer cells.^[^ ^46^ ^]^ In this work, the Au(III) organometallic complex exhibited selectivity to target TXNRD1, as well as potent inhibition against the isolated enzyme and cell extracts. Moreover, the arylation of the CysSec‐dyad was observed in the presence of the complex and reducing conditions, with tandem‐MS identifying the simultaneous bis‐arylation of the Cys and Sec residues. Despite the complex selectively targeting TXNRD1, the protein itself was not regulated; however, three selenoproteins were significantly downregulated: glutathione peroxidase 1 (GPX1) and 4 (GPX4), and adenylyltransferase SelO (SELO), which could be a result of the reduced selenium levels after treatment with [Au(C^NOx^N)Cl_2_].^[^ ^46^ ^]^

Interestingly, selenium arylation of GPX1 has also been reported with the complex, [Au(C^CH2^N)Cl_2_] (Figure 1) and bovine GPX1 containing 1 Sec and 5 Cys residues.^[^ ^47^ ^]^ De Paiva and coworkers incubated the Au(III) complex with GPX1 from *Bos Taurus* erythrocytes in biologically compatible conditions (2 mM ammonium acetate aqueous buffer, pH 7.0, 37 °C), showing complete arylation of both the Sec residue and all 5 Cys residues over 24 h.^[^ ^47^ ^]^

Additionally, in a recent preprint from the same group, the preference of [Au(C^CH2^N)Cl_2_] and [Au(C^CO^N)Cl_2_] (Figure 1) to arylate diphenyl diselenide ((Ph‐Se)_2_) rather than an excess of diphenyl disulfide ((Ph‐S)_2_) was described, even without the presence of reducing agents.^[^ ^48^ ^]^ This was also demonstrated in a cellular context with preferential targeting of the wild‐type (WT) GPX4 selenoprotein in A375 cells rather than the U46C mutant with the Sec residue replaced with a Cys,^[^ ^48^ ^]^ highlighting a strong chemoselective preference of these Au complexes towards selenium.

Interestingly, looking back at Cys‐selective arylation, Gukathasan et al. investigated the systematic tuning of the cyclometalated Au(III) complexes for Cys‐selective arylation and ligand‐directed bioconjugation.^[^ ^49^ ^]^ Their study explored a library of gold complexes of the type [Au(C^CO^N)X_2_] with varying ancillary ligands and provided X‐ray crystallographic evidence of Au(III)‐Cys adduct formation. A key finding was that Au(III) complexes could be fine‐tuned to modulate reactivity, allowing for selective targeting of proteins such as mutant KRAS, a key driver in oncogenic signaling. Structural modifications of the Au(III) complexes led to improved selectivity and efficiency in protein labeling,^[^ ^49^ ^]^ demonstrating the potential for gold‐mediated bioorthogonal modifications in therapeutic applications. These findings provide an essential synthetic framework that complements the biological applications discussed earlier, reinforcing how rational ligand tuning enables greater precision in targeting redox‐active proteins.

**Figure 4:**
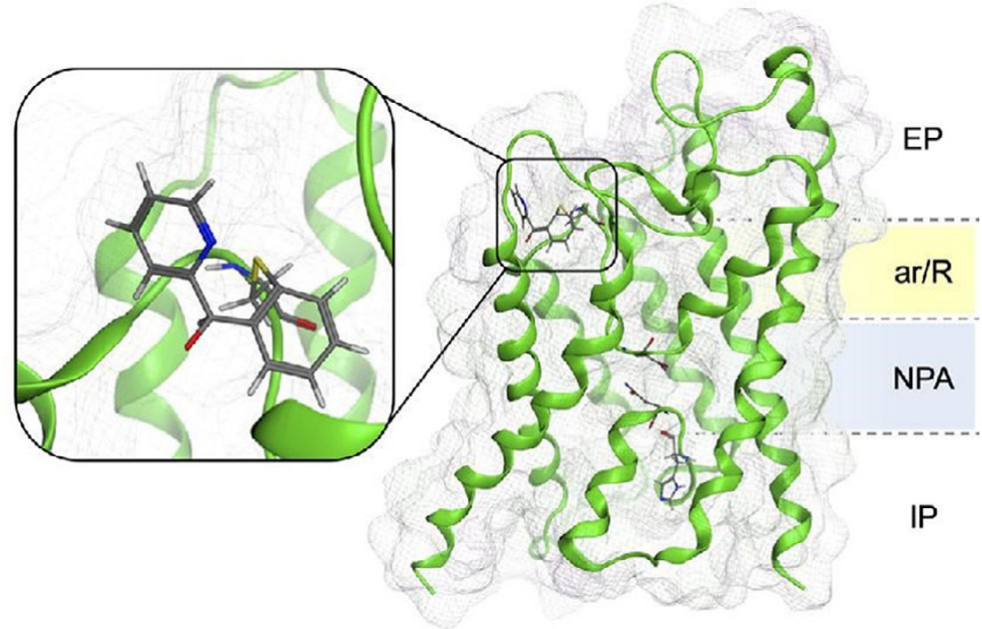
A side‐view of the human AQP10 monomer. Inset: zoom image of the C^CO^N ligand covalently bound to Cys209 after reductive elimination. Figure generated using MOE software. Image from Pimpão, Catarina; Wragg, Darren, Mechanisms of irreversible aquaporin‐10 inhibition by organogold compounds studied by combined biophysical methods and atomistic simulations, Metallomics, 2021, 13, 9, mfab053, Reproduced with permission.^[50]^ Copyright 2021, Oxford University Press.

Beyond enzymatic inhibition, cyclometalated Au(III) C^N complexes have shown promise in modulating transmembrane proteins, particularly in the case of aquaporins (AQPs), which regulate cellular water and glycerol transport. Pimpão et al. investigated the inhibition of AQP10 by a series of cyclometalated Au(III) complexes by wet lab experiments and metadynamic simulations, demonstrating that [Au(C^CO^N)Cl_2_] mediated‐Cys arylation at Cys209 leads to global conformational changes, effectively blocking glycerol permeability (**Figure** **4**). This alteration prevents normal osmotic regulation in cells, potentially impacting metabolic and homeostatic functions.^[^ ^50^ ^]^ This study was further expanded by Da Silva et al. who examined AQP3 inhibition in melanoma cells, where three different gold compounds [Au(C^CO^N)Cl_2_], [Au(C^CH2^N)Cl_2_] and [Au(C^NH^N)Cl_2_] (Figure 1) impaired H_2_O_2_ transport, disrupting cellular redox balance and ultimately inhibiting cancer cell adhesion, proliferation, and migration.^[^ ^51^ ^]^ AQP3, a known peroxiporin, facilitates hydrogen peroxide diffusion across membranes and is frequently overexpressed in melanoma cells. The gold complexes’ ability to selectively block AQP3 activity highlights a potential therapeutic strategy in targeting cancer progression through the modulation of oxidative stress.^[^ ^51^ ^]^ The overlap in targeting redox‐sensitive pathways between enzyme inhibition and transmembrane protein modification illustrates how Au(III) complexes can be strategically tailored to different cellular contexts, making them adaptable tools in therapeutic development.

Collectively, these studies illustrate the broad applicability of cyclometalated Au(III) complexes, spanning antimicrobial, anticancer, and biochemical tool development. The shared mechanistic themes of Cys arylation position these compounds as unique and versatile candidates in the development of targeted therapeutics and biochemical probes.

## 6 Cysteine Arylation In Vivo

**Figure 5:**
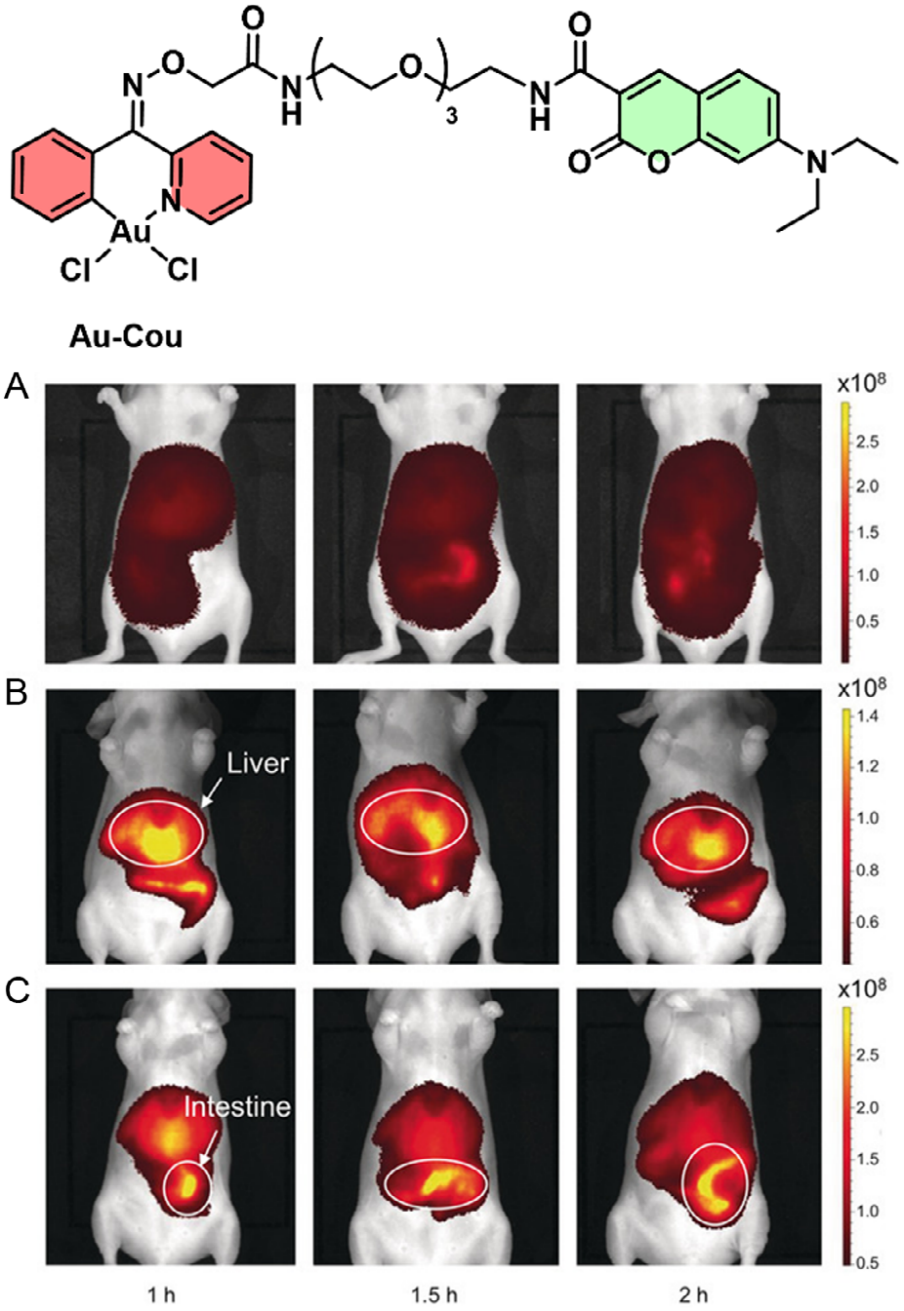
Structure of Au(III) conjugated organometallic complex Au‐Cou. Time‐course imaging of BALB/c nude mice (abdominal view) with liver and intestine‐selective fluorescence labeling treated with A) glycoalbumin (Sia) followed by Cy7.5‐OProp, B) Glyco‐Au (Sia) followed by Cy7.5‐OProp, and C) Glyco‐Au(Gal) followed by Cy7.5‐OProp. Adapted with permission from Tanaka et al. Angewandte Chemie International Edition, 2017, John Wiley and Sons.^[^ ^52^ ^]^

The potential of Au(III) complexes to perform selective chemical modifications in biological environments has also been demonstrated in live animal models. The study by Tsubokura et al. employed glycoalbumin–Au(III) conjugates to catalyze amide bond formation with nearby proteins in live mice, using a Au(III) catalyst (Au‐Cou in **Figure** **5**).^[^ ^52^ ^]^ In this study, organ‐targeting glycoalbumins (α(2‐6)‐disialoglycoalbumin (Sia) to target the liver and galactosylglycoalbumin (Gal) to target the intestine) formed conjugates with coumarin‐Au(III) complexes in the body due to the well‐known affinity of coumarin with the binding pocket of albumin. Therefore, the coumain‐Au(III) complex was first administered intravenously to the mouse, where it rapidly accumulated in the specific organs due to the albumin conjugate formation. A fluorescent propargyl ester probe (Cyanine7.5‐OProp) was then introduced to visualize catalytic transformations, demonstrating that the Au(III)‐catalyzed amide bond formation with surface amino residues was highly selective as can be seen in Figure 5A–C, with no fluorescence observed without the Au complex (Figure 5A), and localized fluorescence in the liver (Figure 5B) and intestine (Figure 5C) with the targeted Au complexes (Glyco‐Au (Sia) and Glyco‐Au (Gal), respectively), over time. Post‐mortem tissue analysis using mass spectrometry and fluorescence imaging confirmed that the gold‐mediated reactions occurred primarily on lysine‐rich surface proteins, ensuring localized reactivity. Additionally, the reaction efficiency was measured through comparative fluorescence quantification in different tissues, proving that organ‐specific targeting was achieved with minimal off‐target modifications.^[^ ^52^ ^]^

Beyond protein labeling, gold complexes have also been utilized in targeted cancer therapy. The work by Vong et al. introduced selective cell tagging (SeCT) therapy, wherein Au(III)‐catalyzed cell surface tagging was employed to selectively modify cancer cells in vivo.^[^ ^53^ ^]^ Their approach involved the design of glycosylated artificial metalloenzyme (GArM) complexes, which facilitated the specific accumulation of the aforementioned Au‐Cou (Figure 5) in tumor tissues. In a mouse xenograft model of human carcinoma, these gold complexes were injected intravenously and allowed to circulate before administration of tagging reagents such as integrin‐blocking cyclic Arg‐Gly‐Asp (cRGD) peptides or cytotoxic doxorubicin derivatives. Histological analyses of tumor biopsies revealed that these modifications occurred predominantly at the tumor periphery, where the highest integrin expression was observed. Cellular assays confirmed that the tagged cancer cells exhibited significantly reduced adhesion to the extracellular matrix, impairing tumor invasion. Mice receiving SeCT therapy showed a notable delay in tumor onset and a reduction in tumor volume over time compared to control groups. Further immunohistochemical staining of tumor sections confirmed that gold‐tagged cells underwent apoptosis at a higher rate, reinforcing the hypothesis that gold‐mediated modifications directly disrupt cancer cell survival mechanisms.^[^ ^53^ ^]^

Further expanding on gold‐catalyzed therapeutic applications, Tanaka and coworkers explored in vivo metal‐catalyzed SeCT therapy using a proapoptotic peptide‐functionalized with the same catalyst (Au‐Cou, Figure 5).^[^ ^54^ ^]^ Their study involved the synthesis of a modified peptide that, upon conjugation with gold (or ruthenium) ions, would covalently attach to tumor cell membranes and induce apoptosis. A dual‐functional system was designed where the catalyst‐mediated attachment of the peptide was followed by its enzymatic activation, triggering programmed cell death. In a murine tumor model, mice were injected with the catalyst‐bearing peptide conjugate, followed by a triggering agent to initiate apoptosis. The efficacy of this approach was assessed by monitoring tumor regression using bioluminescence imaging and histopathological examination of treated tissues. Tumors in mice treated with the gold‐peptide system showed a significant reduction in metabolic activity as measured by PET scans, and tumor sections stained with apoptosis markers revealed extensive cell death. Importantly, the study also examined systemic toxicity by assessing biomarkers for liver and kidney function, confirming that treatment with gold catalysts resulted in minimal adverse effects compared to conventional chemotherapy. Survival studies demonstrated a substantial increase in lifespan for mice receiving this gold‐based treatment,^[^ ^54^ ^]^ highlighting its potential for clinical translation.

## 7 Summary and Outlook

Au(III) organometallic complexes have emerged as versatile catalysts for cross‐coupling reactions in biological environments, with a particular focus on C—S/Se bond formation as a key bioconjugation strategy. Unlike traditional transition‐metal cross‐coupling reactions that often require organic solvents and harsh reaction conditions, organo‐Au(III)‐catalyzed C–S bond formation proceeds efficiently in aqueous media and under physiologically relevant conditions, making it particularly attractive for biological applications. The ability of these Au(III) complexes to selectively arylate cysteine residues has positioned them as powerful tools for site‐selective biomolecular modifications, enabling precise enzyme inhibition, protein labeling, and bioorthogonal transformations.

In particular, the incorporation of cyclometalating ligands has been shown to enhance stability, reactivity, and specificity, yielding finely tuned reactive systems. Various studies have demonstrated how ligand modifications can modulate the rate and selectivity of cysteine or selenocysteine arylation, providing new opportunities for tailoring Au(III) cyclometalated complexes toward targeted biological applications. Beyond their use as bioconjugation reagents, Au(III) organometallic complexes have been investigated for their ability to modulate enzymatic functions, particularly by targeting thiol‐containing proteins such as thioredoxin reductase (TrxR) and aquaporins. Their unique electrophilic properties allow them to interfere with different cellular processes and redox homeostasis, further expanding their potential beyond labeling toward therapeutic intervention.

In addition to C–S/Se cross‐coupling, other Au(III)‐catalyzed cross‐coupling strategies have broadened the scope of biomolecular modifications. For instance, Ko et al. demonstrated that Au(III)‐mediated alkynylation proceeds efficiently in aqueous conditions, expanding the applicability of gold catalysts for protein functionalization and site‐selective labeling.^[^ ^55^ ^]^ Similarly, the Wong group introduced a visible‐light‐induced Au(III) alkynylation, highlighting how photoactivation can be used to control peptide modifications.^[^ ^56^ ^]^ A similar approach has also been reported by Jiang et al. for the light‐triggered prodrug activation in the presence of alkyl‐Au(III) complexes catalyzing β‐hydride elimination reaction.^[^ ^57^ ^]^ Additionally, Chang et al. demonstrated that cyclometalated Au(III) complexes could induce immunogenic cell death (ICD) in malignant pleural mesothelioma, offering a catalysis‐driven approach to immune modulation in cancer therapy.^[^ ^58^ ^]^ While distinct from cysteine arylation, these studies further illustrate the versatility of Au(III) complexes in catalyzing bioorthogonal transformations, reinforcing their role in chemical biology, where Au(III) complexes can be rationally designed to influence cellular pathways, making them also promising candidates for metallodrug development.

Taken together, these studies underscore the dual role of Au(III) organometallic complexes as both selective cross‐coupling catalysts and innovative therapeutic agents. Future research efforts should focus on optimizing ligand design and reaction conditions to further enhance the efficiency, stability, and selectivity of organo‐Au(III)‐catalyzed cross‐coupling reactions, paving the way for new applications in both fundamental biochemistry and precision medicine.

## Conflict of Interest

The authors declare no conflict of interest.
